# Deoxynucleoside Salvage in Fission Yeast Allows Rescue of Ribonucleotide Reductase Deficiency but Not Spd1-Mediated Inhibition of Replication

**DOI:** 10.3390/genes8050128

**Published:** 2017-04-25

**Authors:** Oliver Fleck, Ulrik Fahnøe, Katrine Vyff Løvschal, Marie-Fabrice Uwamahoro Gasasira, Irina N. Marinova, Birthe B. Kragelund, Antony M. Carr, Edgar Hartsuiker, Christian Holmberg, Olaf Nielsen

**Affiliations:** 1 Cell Cycle and Genome Stability Group, Department of Biology, University of Copenhagen, DK-2200 Copenhagen, Denmark; o.fleck@bangor.ac.uk (O.F.); ulrik@sund.ku.dk (U.F.); katrine.lovschal@bio.ku.dk (K.V.L.); incheto0505@gmail.com (I.N.M.); cholm@bio.ku.dk (C.H.); 2 North West Cancer Research Institute, Bangor University, Bangor, Gwynedd LL57 2UW, UK; Marie.Gasasira@sussex.ac.uk (M.-F.U.G.); e.hartsuiker@bangor.ac.uk (E.H.); 3 Structural Biology and NMR Laboratory, Department of Biology, University of Copenhagen, DK-2200 Copenhagen, Denmark; bbk@bio.ku.dk; 4 Genome Damage and Stability Centre, University of Sussex, Brighton BN1 9RQ, UK; a.m.carr@sussex.ac.uk

**Keywords:** DNA replication, checkpoints, ribonucleotide reductase, PCNA, CRL4^Cdt2^, intrinsically disordered proteins, deoxynucleotide salvage, fission yeast

## Abstract

In fission yeast, the small, intrinsically disordered protein S-phase delaying protein 1 (Spd1) blocks DNA replication and causes checkpoint activation at least in part, by inhibiting the enzyme ribonucleotide reductase, which is responsible for the synthesis of DNA building blocks. The CRL4^Cdt2^ E3 ubiquitin ligase mediates degradation of Spd1 and the related protein Spd2 at S phase of the cell cycle. We have generated a conditional allele of CRL4^Cdt2^, by expressing the highly unstable substrate-recruiting protein Cdt2 from a repressible promoter. Unlike Spd1, Spd2 does not regulate deoxynucleotide triphosphate (dNTP) pools; yet we find that Spd1 and Spd2 together inhibit DNA replication upon Cdt2 depletion. To directly test whether this block of replication was solely due to insufficient dNTP levels, we established a deoxy-nucleotide salvage pathway in fission yeast by expressing the human equilibrative nucleoside transporter 1 (hENT1) and the Drosophila deoxynucleoside kinase. We present evidence that this salvage pathway is functional, as 2 µM of deoxynucleosides in the culture medium is able to rescue the growth of two different temperature-sensitive alleles controlling ribonucleotide reductase. However, salvage completely failed to rescue S phase delay, checkpoint activation, and damage sensitivity, which was caused by CRL4^Cdt2^ inactivation, suggesting that Spd1—in addition to repressing dNTP synthesis—together with Spd2, can inhibit other replication functions. We propose that this inhibition works at the point of the replication clamp proliferating cell nuclear antigen, a co-factor for DNA replication.

## 1. Introduction

Proliferating cell nuclear antigen (PCNA) is an essential co-factor for DNA polymerases during DNA replication in eukaryotes. It forms a ring-shaped homotrimer that encircles the double helix and tethers polymerases to DNA, thereby increasing their rate of processivity. PCNA also serves as a platform for recruiting numerous other proteins to DNA, and hence is important for the metabolism of DNA and chromatin, during replication and repair. Most partner proteins bind PCNA via a conserved sequence called the PCNA-interacting protein-box (PIP-box), which associates with a hydrophobic pocket on the front face of the PCNA ring. The consensus PIP-box has the structure Q-x-x-Φ-x-x-Ω-Ω, in which Φ is a hydrophobic amino acid (L, V, I, or M) and Ω is an aromatic residue (Y or F). However, many PCNA interacting proteins have degenerate PIP-box sequences [1].

Since PCNA is a trimer, it can bind more than one PIP-box protein at a time. Consequently, PCNA has been proposed to function as a “tool belt” that can orchestrate the sequential recruitment of enzymes e.g., during maturation of Okazaki fragments [2]. In addition, a specialized E3 ubiquitin ligase called CRL4^Cdt2^ is dedicated to the degradation of proteins bound to PCNA, see [3]. A large number of substrates have been identified for PCNA-targeted degradation, including the cyclin-dependent kinase (CDK) inhibitor p21, the replication licensing factor Cdt1, and the histone methyltransferase Set8 [3]. Ubiquitylation-mediated degradation of these substrates occurs only when they associate with chromatin bound PCNA during S phase, or after DNA damage occurs. The proteins degraded through this pathway harbor a special version of the PIP-box, called a PIP-degron: Q-x-x-Φ-T-D-Ω-Ω-x-x-x-B, where B is a basic residue (K or R). CRL4^Cdt2^ mediated protein turnover at PCNA is thought to contribute to the orderly orchestration of replication and repair events. However, binding of p21 to PCNA can also directly inhibit replication [4].

In fission yeast, cells defective in the CRL4^Cdt2^ E3 ubiquitin ligase rely on the Rad3^ATR^ (ATR: ataxia telangiectasia- and Rad3-related) checkpoint for survival. Curiously, the checkpoint activation is due to the untimely accumulation of a single CRL4^Cdt2^ substrate, a small intrinsically-disordered protein (IDP) called S-phase delaying protein 1 (Spd1) [5,6,7,8,9,10]. Spd1 was originally identified in a screen for proteins that inhibited replication when overexpressed [11]. The CRL4^Cdt2^ E3 ubiquitin ligase is activated by transcriptional induction of the Cdt2 substrate recruiting factor, which becomes expressed prior to S phase by the MluI cell cycle box (MCB) transcription complex [9]. Consequently, Spd1 is degraded during DNA replication in wild-type cells, whereas CRL4^Cdt2^ defective cells undergo S phase in the presence of Spd1, which gives rise to severe S-phase stress—cells accumulate during S phase and their survival relies notably on the activation of the Rad3 checkpoint. Furthermore, such cells are hypersensitive to DNA-damaging agents, they are defective in double-strand break (DSB) repair by homologous recombination, they display a more than 20-fold increase in spontaneous mutation rates, and they are also unable to undergo pre-meiotic S phase [5,7,12]. The requirement for a functional Rad3 pathway and the defects in recombination and pre-meiotic S-phase are all fully suppressed by deleting the *spd1* gene, suggesting that these phenotypes are caused by Spd1 mediated interference with key replication functions.

The first clue towards a replication target of Spd1 came from the observation that overexpression of the *suc22* gene could relieve the checkpoint activation caused by Spd1 accumulation [5]. The *suc22* gene encodes the small subunit of the enzyme ribonucleotide reductase (RNR) responsible for the rate-limiting step in the synthesis of deoxynucleotide triphosphates (dNTPs). Furthermore, it was found that Spd1 sequesters Suc22 in the nucleus, away from the large pan-cellular RNR subunit Cdc22, thereby reducing the cytosolic level of active RNR complexes [5]. However, mutants in *spd1* that were defective in nuclear localization of Suc22, but still required *rad3* in a CRL4^Cdt2^ mutated background, were subsequently identified, suggesting that checkpoint activation is not (or only in part) due to nuclear localization of Suc22 [8]. Consistent with Spd1 inhibiting RNR, deletion of the *spd1* gene leads to a twofold increase in cellular dNTP pools [13]. Also, Spd1 can inhibit the enzymatic activity of RNR, and binds to both subunits in vitro [8,14].

However, several observations have challenged the view that Spd1 causes checkpoint activation by inhibiting RNR. First, while overexpression of Suc22 can suppress the checkpoint activation caused by Spd1 accumulation, overexpression of the large RNR R1 subunit Cdc22 fails to do so [15]. Since Suc22 directly binds to Spd1 [8] (B.B.K., unpublished observations), suppression may—in addition to boosting RNR activity—function by titrating Spd1 away from another target. Strikingly, cells harboring the *cdc22-D57N* mutation, defective in dATP-mediated feedback inhibition of RNR, have a 2–5-fold increase in cellular dNTP pools. Yet, when Spd1 accumulation is induced in this *cdc22-D57N* background by CRL4^Cdt2^ inactivation, the checkpoint is still required, even though dNTP pools are higher than the wild-type levels [13].

The fission yeast genome encodes a second Spd1-related CRL4^Cdt2^-targeted IDP, called Spd2 [16]. Overexpression of Spd2 can also delay S phase, but Spd2 does not appear to regulate dNTP pools. Interestingly, on their own, *cdc22-D57N* or *Δspd2* only weakly rescue the Rad3 requirement of CRL4^Cdt2^-deficient cells. However, in the *cdc22-D57N Δspd2* double mutant, the checkpoint requirement is fully suppressed, similar to the case of *spd1* deletion [16]. This observation suggests that Spd1 can cause checkpoint activation via both deoxynucleotide-dependent and -independent mechanisms, and that Spd2 only contributes to the latter.

In the present study we have developed a conditional *cdt2* allele that allows us to study the immediate effects of Spd1 and Spd2 accumulation. We show that Cdt2 depletion causes a strong inhibition of DNA replication that is dependent on both Spd1 and Spd2. We also report on the successful expression of a deoxynucleotide salvage pathway in fission yeast, by which we can overcome two mutants in RNR. However, consistent with Spd1 having other targets than RNR, nucleotide salvage was completely unable to relieve the replication problems and checkpoint activation induced by Spd1 accumulation.

## 2. Materials and Methods

### 2.1. Molecular and Genetic Procedures

The *Schizosaccharomyces pombe* strains used in the present study are listed in Supplementary Table S1. Standard genetic procedures were performed according to [17]. The *cdt2* open reading frame (ORF), including its stop codon, was recombined into the vector pDUAL-FFH41 [18] using Gateway technology (Invitrogen, Waltham, MA, USA). The resulting plasmid was digested with *Not*I and integrated at the *leu1* locus, rendering Cdt2 expression repressible by thiamine. The *Drosophila melanogaster* gene encoding deoxyribonucleoside kinase (DmdNK) [19] under control of the fission yeast *adh* promoter, was integrated into the *S. pombe* genome, replacing *ura4*. Subsequently, the human equilibrative nucleoside transporter (hENT1) gene [20] under control of the *adh* promoter, coupled to a nourseothricin (*natMX*) resistance marker, was integrated adjacent to *DmdNK*.

### 2.2. Physiological Experiments and Cell Biology

Cells were grown at 30 °C (unless otherwise stated) in minimal sporulation liquid (MSL) media [21] to a concentration of 5 × 10^6^ cells/mL. Thiamine was added to a concentration of 5 µg/mL, when indicated. For spot test survival assays, 7 µL of 10-fold serial dilutions (starting with 10^7^ cells/mL) were spotted on MSA plates (MSL with 2% agar) with indicated additives, and incubated 2–4 days at the indicated temperature. Cell cycle synchronization at G1 by nitrogen starvation in the presence of M-factor, and analysis of cell-cycle distribution by flow cytometry, were performed as described by [22]. FACS data were processed by the program FlowJo (FlowJo, Asland, OR, USA). Bimolecular fluorescence complementation (BiFC) was performed with the same constructs as described in [6]. Cds1 kinase assays and dNTP pool measurements were performed as previously described [13]. 5-ethynyl-2’-deoxyuridine (EdU) was added to 10 µM to cells growing exponentially at 30 °C in MSL; after 20 min, the cells were fixed in 70% ethanol. Incorporated EdU was coupled to Alexa Flour 545 azide as described [23]. Fluorescence and Nomarski microscopy was performed on a Zeiss Axio Imager platform (Zeiss, Jena, Germany).

## 3. Results

### 3.1. Generation of a Conditional CRL4^Cdt2^ Mutant

Accumulation of Spd1 and Spd2 in cells with defective CRL4^Cdt2^ causes slow progression of the S phase and activation of the Rad3 checkpoint, which also becomes essential for cell survival. However, since CRL4^Cdt2^ defective cells have been deficient for many generations, it is difficult to discriminate between immediate and compensatory effects. In order to circumvent this problem, we constructed a conditional CRL4^Cdt2^ allele that could be inactivated within a single cell cycle. The E3 substrate recruiting protein Cdt2 has been reported to exhibit rapid turnover with a half-life of 10–15 min throughout the cell cycle [9], making repression of its transcription a good choice for fast down-regulation of CRL4^Cdt2^ function. We therefore expressed Cdt2 from the thiamine repressible *nmt41* promoter [24] integrated at the *leu1* locus in a *Δcdt2* background. We refer to this allele as *cdt2^TR^* (for thiamine repressible).

**Figure 1:**
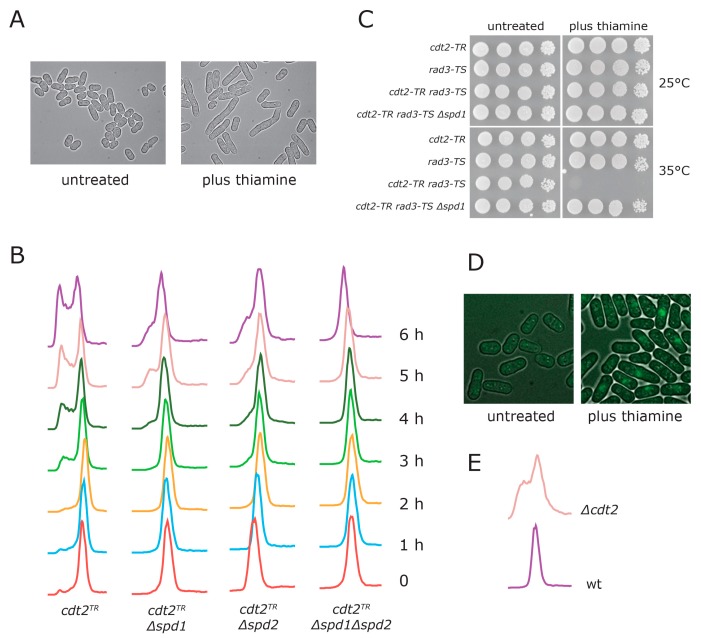
Characterization of the *cdt2^TR^* allele. (**A**) *cdt2^TR^* cells were grown in minimal sporulation liquid (MSL) medium at 30 °C. The culture was divided in two, thiamine was added to the indicated culture, and pictures were taken with Nomarski optics after 12 h; (**B**) *cdt2^TR^* cells of the indicated genotype were grown at 30 °C in MSL medium. At t = 0, thiamine was added to the four cultures. Samples were passed through flow cytometry at hourly intervals, as indicated. The apparent slight drift to the left at early time points in the *Δspd2* strain was due to a DNA staining artefact; (**C**) Serial dilutions of strains with the indicated genotypes were spotted on plates either with or without thiamine, and incubated at the indicated temperature for three days; (**D**) *cdt2^TR^* cells expressing *VN173-pcn1* and *spd1-VC155* [6] were propagated in minimal sporulation liquid (MSL). The culture was divided in two, thiamine was added to the indicated culture, and pictures of yellow fluorescent protein (YFP) fluorescence were taken after four hours; (**E**) DNA content profiles of growing wild type and *Δcdt2* cells.

In the absence of thiamine, cells of this strain appeared normal (Figure 1A), grew with a doubling time similar to wild type (data not shown), and showed a normal DNA content profile (Figure 1B, samples at t = 0), suggesting that the induced level of *cdt2* transcription was sufficient to mediate Spd1 and Spd2 degradation at S phase. However, when we added thiamine to the culture, the cells elongated (Figure 1A), and died in an *spd1*-dependent manner when a temperature sensitive *rad3* allele was inactivated (Figure 1C). Also, we found that when Spd1 and PCNA were tagged with, respectively, the N- and C-terminus of Venus yellow fluorescent protein (YFP), a bimolecular fluorescence complementation (BiFC) signal indicative of interaction between the two proteins was observed following thiamine addition (Figure 1D), as previously reported for *Δcdt2* cells [6]. Flow cytometry showed that cells gradually accumulated in G1 and S phase, indicative of replication problems (Figure 1B, first column). Consistent with *cdt2^TR^* cells forming colonies on plates containing thiamine (Figure 1C), we found that the S phase arrest was only transient (data not shown). Deletion of the *spd1* gene largely suppressed cell cycle arrest (Figure 1B, second column). The transient S phase arrest observed appeared more severe than that which was seen in *cdt2* deleted cells (Figure 1E), presumably because the latter have adapted to the absence of Cdt2 by activating compensatory pathways.

Overexpression of the *spd2* gene can also inhibit replication, but Spd2 does not appear to regulate dNTP pool levels [16]. Spd2 is also degraded via CRL4^Cdt2^-mediated ubiquitylation, but unlike Spd1, Spd2 accumulation in a CRL4^Cdt2^ deficient background does not cause a requirement for the Rad3 checkpoint [16]. However, we found that cells with an *spd2* deletion were, like *Δspd1*, defective in cell cycle arrest upon switching off Cdt2 expression (Figure 1B, third column). These observations demonstrate that the S phase arrest enforced by the inhibition of *cdt2* transcription is dependent on both Spd1 and Spd2 accumulation.

### 3.2. Spd1 Accumulation Causes S Phase Delay

**Figure 2:**
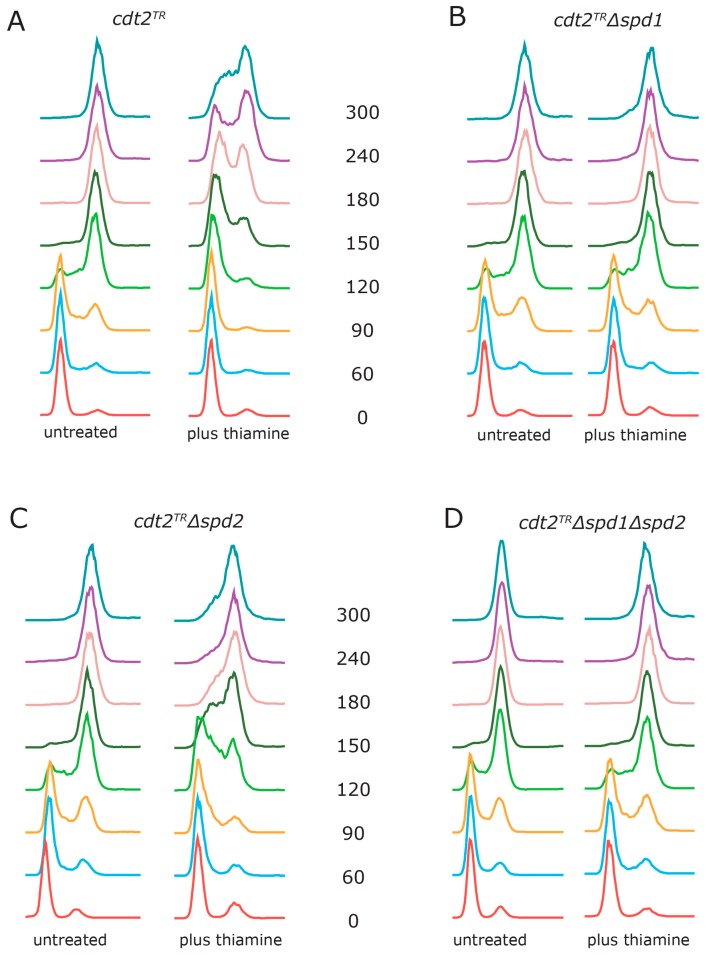
Spd1 accumulation inhibits S phase progression. Cells with the *cdt2^TR^* allele were synchronized in G1 by nitrogen starvation in the presence of the M-factor pheromone [22]. The pheromone was washed away, and the cultures were released into S phase in MSL medium at 30 °C, either with or without thiamine at t = 0. Samples were taken for flow cytometry at the indicated time points (min). Genotypes: (**A**) *cdt2^TR^*; (**B**) *cdt2^TR^ Δspd1*; (**C**) *cdt2^TR^ Δspd2* and (**D**) *cdt2^TR^ Δspd1 Δspd2*.

Next, we wanted to examine how accumulation of Spd1 and Spd2 directly affected S phase progression. In order to do so, we used the recently developed method of M-factor treatment to synchronize cells in G1 [22]. When *cdt2^TR^* cells were released from G1 in the absence of thiamine, cells entered S phase after one hour and completed S phase within three hours (Figure 2A). This is similar to the kinetics observed with wild-type cells [22]. However, if we added thiamine to the cells at the time of release, both entry into, and progression through S phase were substantially delayed (Figure 2A). In fact, the cells had not completed DNA replication at the 300 min time point. These results confirm that we can rapidly downregulate *cdt2* function by repressing its transcription. Deletion of the *spd1* gene completely suppressed the thiamine-induced replication delay (Figure 2B), demonstrating that the replication block was dependent on Spd1 accumulation.

In cells deleted for *spd2*, S phase progression was still substantially delayed by thiamine addition, presumably due to accumulation of Spd1 (Figure 2C). However, the completion of S phase was advanced by approximately two hours relative to *spd2^+^* cells (compare Figure 2C and Figure 2A). When both *spd1* and *spd2* were deleted (Figure 2D), the kinetics were fast, similar to those of *Δspd1* cells (Figure 2B). Taken together, these results show that Spd2 accumulation helps to enforce the replication arrest imposed by Spd1.

### 3.3. Both Branches of the Rad3 Checkpoint Are Involved in Tolerating Replication Problems Caused by Spd1 Accumulation

**Figure 3:**
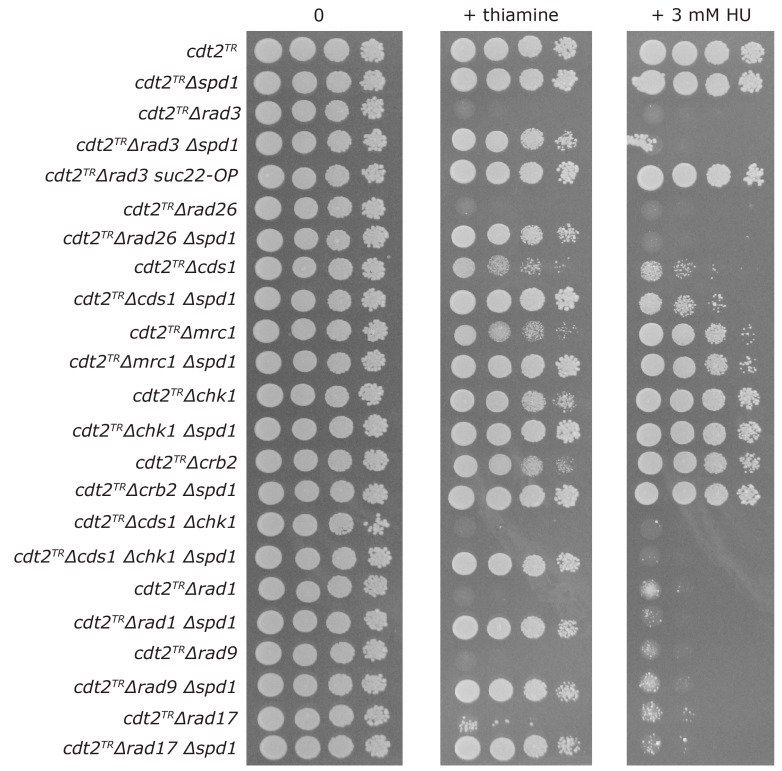
Checkpoint requirement in Spd1 accumulating cells. Cells of the indicated genotype (all salvage background) were spotted onto MSA plates either without or with thiamine, or plates containing 3 mM hydroxyurea (HU). The plates were incubated at 30 °C for three days.

We next defined the extent to which the function of the DNA damage checkpoint, in addition to Rad3, was required for tolerance of Spd1 accumulation during replication. We crossed the conditional *cdt2^TR^* allele into various checkpoint mutant backgrounds, either in the presence or absence of *spd1*, and spotted cells onto plates with or without thiamine (Figure 3). For comparison, we also spotted the cells on plates containing a low concentration of the RNR inhibitor hydroxyurea (HU). In general, there was a good correlation between the checkpoint functions required to tolerate the two S phase inhibitors. However, whereas deletion of the *spd1* gene completely suppressed the sensitivity to checkpoint loss caused by Cdt2 depletion, it had little effect on HU sensitivity, presumably because Spd1 does not inhibit RNR in the absence of thiamine.

The core checkpoint proteins Rad3 and Rad26, as well as the 9-1-1 checkpoint clamp (Rad1 and Rad9) and its loader (Rad17), were all absolutely required for the survival of both HU-treated and Spd1-accumulating cells. Mutants in the two branches of the Rad3 pathway, the replication branch (Cds1 and Mrc1) and the DNA damage branch (Chk1 and Crb2) were both partially sensitive to HU and Spd1 accumulation respectively, indicating that both these branches of the Rad3 pathway can redundantly contribute to tolerance of imposed S phase problems. Consistent with this, we found that the *Δcds1 Δchk1* double mutant was as sensitive as *Δrad3* to both HU treatment and Spd1 accumulation. In agreement with a function of the Cds1-dependent replication branch in tolerating Spd1 accumulation, we found that thiamine addition to *cdt2^TR^* cells caused *spd1*-dependent induction of Cds1 kinase activity (see below).

### 3.4. Establishment of a Deoxynucleotide Salvage Pathway in Fission Yeast

**Figure 4:**
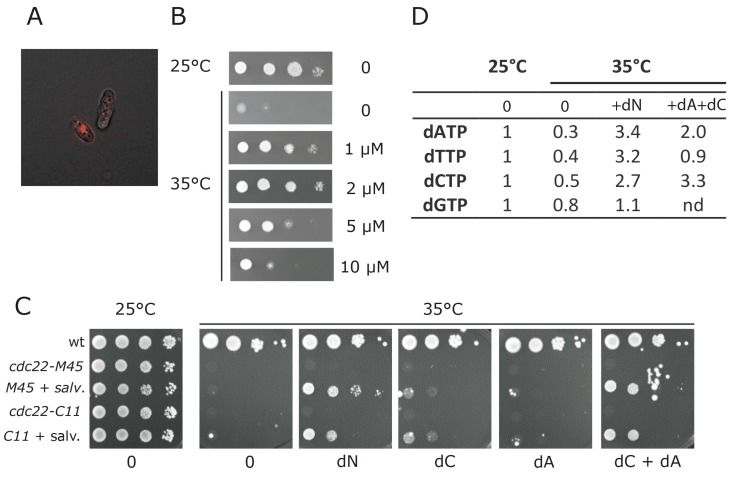
Establishment of a salvage pathway in fission yeast. (**A**) Incorporation of 5-ethynyl-2’-deoxyuridine (EdU) into cells expressing the salvage pathway. The fluorescence image shows a cell that incorporated EdU into its DNA and another cell that did not; (**B**) Growth of *cdc22-M45* cells expressing the salvage pathway at 25 °C or at 35 °C. The plates at 35 °C contained the indicated concentration of dN (an equimolar mixture of dA, dC, dG and dT); (**C**) Growth of wild type cells, or cells harboring two different temperature-sensitive alleles of *cdc22* (M45, C11), either with or without the salvage pathway at, respectively, 25 °C or at 35 °C. The plates at 35 °C contained 2 µM of the indicated deoxynucleoside; (**D**) Deoxynucleotide triphosphate (dNTP) pool measurements of a *cdc22-M45* strain expressing the salvage pathway, grown either at 25 °C, or switched to 35 °C for four hours. When indicated, 4 µM of dN (as defined above) or 4 µM of dA + dC were added to the cultures at the time of temperature shift up. The relative levels of deoxynucleoside triphosphates were normalized to ATP and arbitrarily set to 1 at 25 °C. (nd: not determined).

Spd1 inhibits RNR, and *Δspd1* cells have a two-fold elevation of their dNTP pools [13]. We wanted to directly test whether the inhibition of DNA replication imposed by Spd1 accumulation could be suppressed by restoring dNTP levels. *S. pombe* does not have a deoxynucleotide salvage pathway for uptake and phosphorylation of deoxynucleosides (dN). We therefore engineered fission yeast cells to express the genes for hENT1 and the *D. melanogaster* DmdNK; both from the strong, constitutive *adh1* promoter, and integrated at the *ura4* locus. We chose DmdNK, since it has broad specificity, and can phosphorylate all four deoxynucleosides [25]. Clear fluorescence labeling of cells from accumulation of EdU in DNA was observed in this strain when the nucleoside analogue EdU was added to the culture medium (Figure 4A).

We next evaluated the functionality of this salvage pathway for unmodified DNA building blocks, by testing whether we could bypass the essential function of RNR by adding deoxynucleosides to the culture medium. We first tested whether we could rescue the temperature-sensitive *cdc22-M45* allele of the large subunit of RNR. When crossed into the salvage background, growth of this mutant at the restrictive temperature was restored by addition of deoxyribonucleosides to the culture medium; maximum rescue was observed using a concentration of 2 µM (Figure 4B). In addition to *cdc22-M45*, one other temperature-sensitive allele of *cdc22* called C11 has been isolated [26]. We sequenced both alleles; the *cdc22-M45* mutant encoded an F518S substitution, while the *cdc22-C11* mutant encoded a G591E substitution in Cdc22. When crossed into the salvage background, growth of the *cdc22-C11* mutant at the restrictive temperature was also rescued by addition of 2 µM deoxyribonucleosides to the culture medium (Figure 4C), suggesting that the observed rescue was not allele-specific. Furthermore, for both mutants, rescue was dependent on the presence of the salvage pathway (Figure 4C).

Curiously, rescue of RNR function appeared to depend on the two deoxyribonucleosides dA and dC only (Figure 4C), suggesting that currently unidentified cellular deaminase activities can convert dA and dC into dG and dT respectively. Consistent with the existence of such a conversion pathway, our dNTP concentration measurements indicated that the deoxythymidine triphosphate (dTTP) pool was elevated by extracellular provision of dA and dC in the culture medium, albeit not to the same level as when all four deoxynucleosides (dN) were provided (Figure 4D).

### 3.5. dNTP Salvage Does Not Rescue Spd1 Accumulation

**Figure 5:**
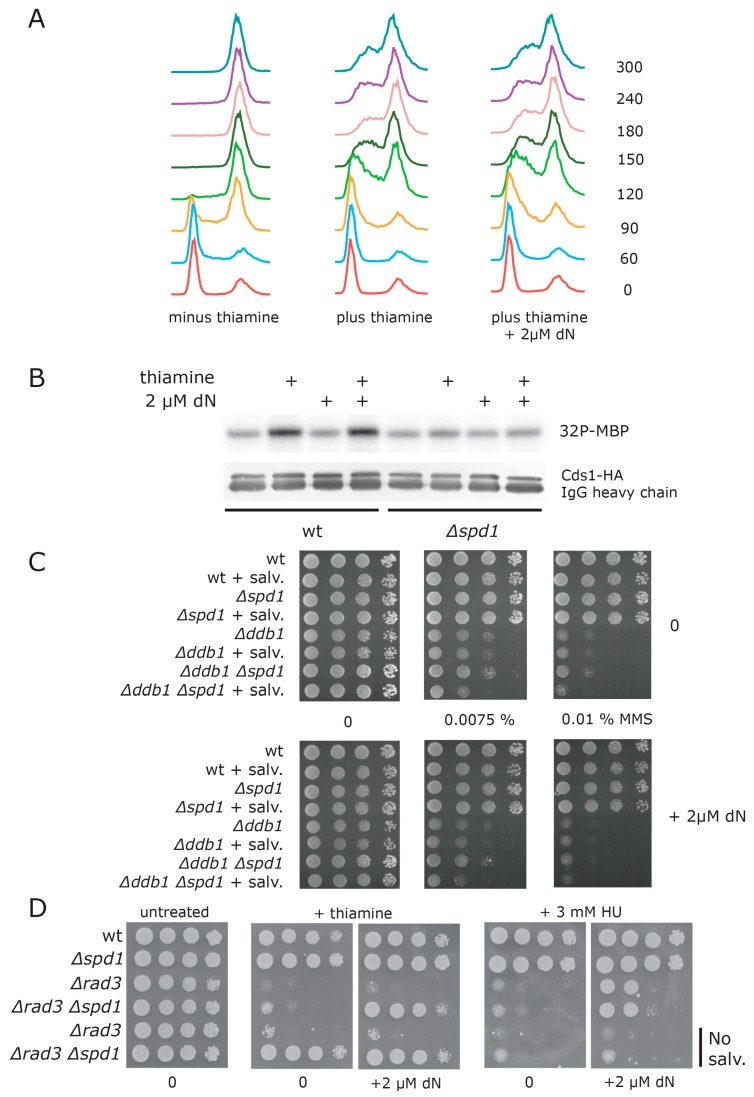
Salvage does not overcome S phase problems caused by Spd1 accumulation. (**A**) FACS profiles of G1 synchronized *cdt2^TR^* cells expressing the salvage pathway. The cells were released in either the absence of presence of thiamine (to induce Spd1 accumulation). The culture in the right panel shows cells that were supplied with 2 µM of dN in addition to thiamine; (**B**) *cdt2^TR^* or *cdt2^TR^ Δspd1* cells expressing the salvage pathway were treated with thiamine and/or 2 µM dN, as indicated. Cells were harvested after four hours, and Cds1 kinase activity against myelin basic protein (MBP) was monitored. Lower panel shows a Western blot of hemagglutinin-tagged Cds1 (HA-Cds1), with immunoglobulin G (IgG) heavy chain serving as a loading control; (**C**) Cells of the indicated genotypes were spotted on plates containing the indicated concentration of methyl methanesulfonate (MMS). “+ salv” means strains expressing the salvage pathway. Plates in the lower panel contain 2 µM of dN; (**D**) Strains of the indicated genotypes were spotted on plates with the indicated supplements (thiamine, 3 mM HU, or 2 µM dN) and grown at 30 °C for three days. All strains contain the *cdt2^TR^* allele. Strains in the two last rows do not express the salvage pathway.

Having established a functional deoxyribonucleoside salvage pathway, we tested whether salvage could overcome the replication problems caused by Spd1 accumulation when Cdt2 is depleted. In these experiments, we used 2 µM of dN, which was the optimal concentration for rescue of the *cdc22* temperature-sensitive mutants. At the time of release, we added dN to the culture medium of G1-synchronized *cdt2^TR^* cells expressing the salvage pathway (Figure 5A). Surprisingly, this did not improve the kinetics of S phase progression in cells with repressed *cdt2* transcription. Furthermore, salvage did not prevent Cds1 kinase activation following Cdt2 depletion (Figure 5B).

Since salvage could not overcome the problems caused by Spd1 accumulation, but could rescue the temperature-sensitive *cdc22* mutations, we were interested to establish whether the salvage could rescue the DNA damage sensitivity of CRL4^Cdt2^ defective *Δddb1* cells. It is proposed that this sensitivity is, in part, caused by cellular dNTP levels being insufficient for repair synthesis [7]. However, as can be seen in Figure 5C, addition of 2 µM dN to *∆ddb1* cells expressing the salvage pathway did not reduce sensitivity to the alkylating agent methyl methanesulfonate (MMS). Increasing the concentration of dN above 2 µM appeared to inhibit growth of *Δddb1* cells (data not shown).

Taken together, these results suggested that Cdt2 depletion at S phase causes problems in addition to its inhibition of dNTP synthesis. To substantiate this conclusion, we directly compare the ability of salvage to rescue the checkpoint activation caused by Spd1 accumulation with that invoked by HU mediated inhibition of RNR, which presumably only affects dNTP synthesis. We investigated the ability of salvage to rescue the killing of *rad3-TS* cells at the restrictive temperature induced by HU addition or *cdt2* depletion (Figure 5D).

Consistent with HU inhibiting RNR only, we found that salvage could improve the survival of the HU-treated *rad3* cells substantially (Figure 5D, right panels, rows 3 and 4; compare 0 µM and 2 µM dN). Curiously, when performing the experiment investigating the effects of Spd1 accumulation, we found that in the salvage background, deletion of *spd1* could no longer rescue the checkpoint requirement of *cdt2*-depleted cells (Figure 5D, second panel, compare rows 3 and 4 with rows 5 and 6). However, when we added 2 µM dN to the salvage strain, rescue by *∆spd1* was restored to a level similar to that observed in cells without the salvage pathway (Figure 5D, third panel, rows 3–6). One explanation for this unexpected observation is that the salvage pathway causes an *spd1*-independent reduction of dNTP pools in cells that can be counteracted by extracellular dN. In any event, as opposed to the case under HU treatment, we did not see any evidence for rescue of Cdt2-depleted *spd1^+^* cells by salvage in this assay (Figure 5D, second and third panels, row 3). In conclusion, this difference between HU and Spd1 is consistent with Spd1 inhibiting other cellular functions in addition to deoxynucleotide synthesis.

## 4. Discussion

Fission yeast cells defective in CRL4^Cdt2^ mediated protein ubiquitylation are challenged at S phase because the Spd1 and Spd2 proteins are not degraded. The thiamine-repressible *cdt2^TR^* allele described in the present report allowed us to study the immediate effects of Spd1 and Spd2 accumulation. When we switched off CRL4^Cdt2^ activity, we saw accumulation of cells in the S phase after three hours (Figure 1B, first column), indicating a rapid effect on cell cycle progression. Interestingly, cells deleted for either *spd1* or *spd2* were compromised for cell cycle arrest, showing that Spd1 and Spd2 both contribute to the S phase arrest observed upon Cdt2 depletion (Figure 1B, second and third column). However, the double mutants showed better S-phase progression after 5–6 h than the two single mutants (Figure 1B, fourth column), indicating that Spd1 and Spd2 on their own can inhibit replication, presumably through a common target. This is consistent with the observation that both Spd1 and Spd2 can block S phase independently of each other when strongly overexpressed [16].

When the ability of G1 synchronized cells to progress through the S phase was scrutinized (Figure 2), we obtained a different result. Here, Spd1 accumulation appeared to be absolutely required for blocking replication (Figure 2B), whereas Spd2 had a relatively small enhancing effect on the arrest. However, the completion of S phase in *Δspd2* cells was advanced by approximately two hours relative to *spd2^+^* cells (compare Figure 2C and Figure 2A), again suggesting that Spd2 can inhibit progression through the S phase. One interpretation of these results is that accumulation of both Spd1 and Spd2 can induce a transient S phase arrest, but maintenance of the arrested state is mostly dependent on Spd1. Presumably, this difference is related to the fact that Spd1 but not Spd2 inhibits dNTP formation [16].

Our analysis of various checkpoint mutants (Figure 3) shows that HU exposure and Spd1 accumulation give rise to S phase problems that can be tolerated by both the replication branch (Cds1 and Mrc1) and the DNA damage branch (Chk1 and Crb2) of the Rad3 pathway. Consequently, the *Δcds1 Δchk1* double mutant is as sensitive as *Δrad3*. Presumably, in the absence of Cds1, exposure to HU or induction of Spd1 accumulation causes fork collapse and subsequent need for the Chk1 sub-pathway [27]. Furthermore, loading of the 9-1-1 checkpoint clamp appears to be essential after both types of replication stress. These observations are consistent with Spd1 exerting its function via the inhibition of RNR (similar to HU).

To directly test whether elevation of dNTP pools could improve S phase delay in Spd1- and Spd2-accumulating cells, we established a salvage pathway in fission yeast. By using the equilibrative hENT1 transporter, we could define the intracellular pools simply by adding a given level of deoxynucleosides to the culture medium. Furthermore, by applying the DmdNK deoxyribonucleoside kinase, we could salvage all four deoxyribonucleotides [28]. This engineered salvage pathway allowed us to rescue two different temperature-sensitive mutants in the *cdc22* gene encoding the essential R1 subunit of RNR (Figure 4C). To our knowledge, this is the first example of salvage of an RNR deficiency in any system. However, it is not clear whether salvage can rescue a strain deleted for both RNR subunits, or whether survival somehow relies on residual RNR functions at the restrictive temperature. Finally, salvage appeared to function with only dA and dC, suggesting that these molecules can be converted into dGTP and dTTP by a pathway involving purine/pyrimidine deaminase activities. The fission yeast genome encodes at least five potential deaminase enzymes, but we have not determined whether any of these are required for salvage by dA and dC.

Our main goal for establishing the salvage pathway was to investigate if restoring dNTP levels through salvage could circumvent the S phase problems caused by Spd1 and Spd2 accumulation. However, salvage neither improved the slow S phase progression (Figure 5A), nor prevented the Cds1 kinase activation (Figure 5B) observed in Cdt2 depleted cells. Moreover, salvage did not suppress the damage sensitivity of CRL4^Cdt2^ defective *Δddb1* cells (Figure 5C). Strikingly, whereas salvage clearly suppressed the killing of *rad3* cells exposed to the RNR inhibiting drug HU, it did not improve the survival of Cdt2-depleted *rad3* cells (Figure 5D). We interpret this observation as evidence for dNTP synthesis-independent inhibition of replication by Spd1. Such an effect likely occurs through interactions with other protein targets, a scenario that is linked to the IDP properties of Spd1, allowing for multi-valency during interactions [29].

**Figure 6:**
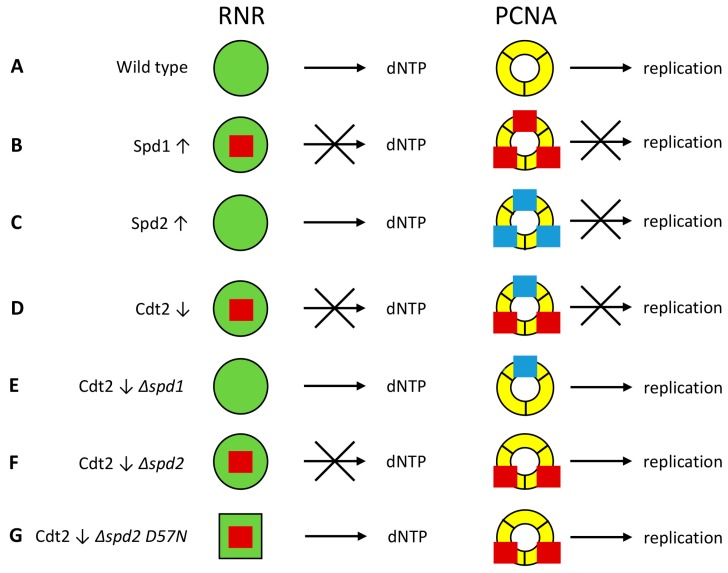
Model for inhibition of DNA replication by Spd1 and Spd2. Spd1 (red squares) can inhibit ribonucleotide reductase (RNR, green circles), while both Spd1 and Spd2 (blue squares) can inhibit replication by binding to proliferating cell nuclear antigen (PCNA, yellow rings). S phase is inhibited if at least one of these two events occurs. (**A**) In wild type cells, both Spd1 and Spd2 are degraded, and hence dNTP production and elongation are not inhibited; (**B**) When Spd1 is overexpressed (↑), it inhibits both processes; (**C**) In cells overexpressing Spd2, only PCNA is inhibited; (**D**) In Cdt2 depleted cells (↓), Spd1 and Spd2 accumulate to a moderate level. Spd1 inhibits RNR, while both Spd1 and Spd2 are required to raise the concentration to a level where inhibition of PCNA can occur; (**E**) Deletion of *spd1* relieves both types of repression; (**F**) When *spd2* is deleted in Cdt2-depleted cells, repression of PCNA is lifted, but Spd1 still inhibits RNR; (**G**) The *cdc22-D57N* mutation changes the RNR configuration (green square) so that it can no longer be inhibited by dATP through negative feedback. Hence, sufficient amounts of dNTPs are formed even in the presence of Spd1 inhibition.

But what is this other target of Spd1? Human p21 is a CRL4^Cdt2^-targeted IDP that can inhibit replication by binding to PCNA [4], and heterologous expression of p21 causes checkpoint activation at PCNA in fission yeast [30]. We propose that Spd1, together with Spd2, can similarly inhibit progression of the replication fork by binding to PCNA (Figure 6). Spd1 can bind to PCNA [6] (Figure 1D), and Spd2 is most similar to Spd1 in the HUG domain containing the PIP degron that binds to PCNA [16]. Furthermore, Spd2 also appears to bind PCNA in vitro (B.K.B., data not shown). Hence, when overexpressed from the strong *nmt1* promoter, both Spd1 (Figure 6B) and Spd2 (Figure 6C) can block replication independent of each other [16]. However, when CRL4^Cdt2^ is downregulated, we speculate that Spd1 and Spd2 accumulate to an intermediate level, such that both proteins are required for inhibition of PCNA (Figure 6D). Deletion of *spd1* hence relieves inhibition from both RNR and PCNA, and therefore suppresses the checkpoint activation caused by CRL4^Cdt2^ inactivation (Figure 6E). On the other hand, when *spd2* is deleted, it is only the PCNA inhibition that is relieved; Spd1 will still inhibit RNR (Figure 6F). Consequently, it is also necessary to elevate dNTP pools by means of the *cdc22-D57N* mutation in order to suppress checkpoint activation in CRL4^Cdt2^ defective *Δspd2* cells [16] (Figure 6G). At this stage it is unclear whether Spd1 and Spd2 cause checkpoint activation merely by binding to PCNA, or whether they perturb the recruitment of other replication factors. Resolving this issue will require detailed studies of the interactions between Spd1, Spd2 and PCNA.

## Supplementary Materials

The following are available online at www.mdpi.com/2073-4425/8/5/128/s1. Table S1: Strain list.
