# Supplementary material for: Deoxynucleoside Salvage in Fission Yeast Allows Rescue of Ribonucleotide Reductase Deficiency but Not Spd1-Mediated Inhibition of Replication
Source: Genes (Basel). 2017 Apr 25;8(5):128. doi: 10.3390/genes8050128 (PMC5448002; doi:10.3390/genes8050128)
Supplement: Supplementary file 1 [file genes-08-00128-s001.pdf]

Table S1. List of strains used in this study.

EA1	*h*$^-$ *Δddb1::kanMX ura4::adh::dmdNK-natMX-adh::hENT1 ura4-aim*
EA5	*h*$^-$ *Δspd1::hphMX ura4::adh::dmdNK-nat-adh::hENT1 ura4-aim*
EA7	*h*$^-$ *Δddb1::kanMX Δspd1::hphMX ura4::adh::dmdNK-natMX-adh::hENT1 ura4-aim*
Eg545	*h*$^+$ *Δmat2,3*
Eg2261	*h*$^+$ *Δcdt2::ura4*$^+$ *ura4-D18 leu1-32 ade6-704*
Eg2352	*h*$^-$ *leu1-32 ura4-D18*
Eg2664	*h*$^+$ *cdc22-M45*
Eg2686	*h*$^+$ *rad3-TS*
Eg3027	*h*$^-$ *leu1*$^+$*::nmt41-cdt2 Δcdt2::ura4*$^+$ *ura4-D18*
Eg3055	*h*$^+$ *leu1*$^+$*::nmt41-cdt2 Δcdt2::ura4*$^+$ *ura4-D18*
Eg3056	*h*$^+$ *leu1*$^+$*::nmt41-cdt2 Δcdt2::ura4*$^+$ *ura4-D18 rad3-TS*
Eg3178	*h*$^+$ *leu1*$^+$*::nmt41-cdt2 Δcdt2::ura4*$^+$ *ura4-D18*
Eg3188	*h*$^+$ *leu1*$^+$*::nmt41-cdt2 Δcdt2::ura4*$^+$ *ura4::adh-dmdNK-natMX-adh-hENT*
Eg3192	*h*$^+$ *leu1*$^+$*::nmt41-cdt2 Δcdt2::ura4*$^+$ *ura4::adh-dmdNK-natMX-adh-hENT Δspd1::hphMX*
Eg3194	*h*$^+$ *leu1*$^+$*::nmt41-cdt2 Δcdt2::ura4*$^+$ *ura4-D18 Δspd1::hphMX*
Eg3302	*h*$^-$ *cdc22-C11*
Eg3439	*h*$^+$ *leu1*$^+$*::nmt41-cdt2 Δcdt2::ura4*$^+$ *ura4-D18 Δspd1::hphMX rad3-TS*
Eg3455	*h*$^+$ *leu1*$^+$*::nmt41-cdt2 Δcdt2::ura4*$^+$ *Δspd2::kanMX*
Eg3457	*h*$^+$ *leu1*$^+$*::nmt41-cdt2 Δcdt2::ura4*$^+$ *Δspd1::hphMX*
Eg3459	*h*$^+$ *leu1*$^+$*::nmt41-cdt2 Δcdt2::ura4*$^+$ *Δspd1::hphMX Δspd2::kanMX*
Eg3490	*h*$^+$ *leu1*$^+$*::nmt41-cdt2 Δcdt2::ura4*$^+$
Eg3503	*h*$^-$ *cdc22-M45 ura4::adh-dmdNK-naMXt-adh-hENT ura4-aim*
Eg3504	*h*$^-$ *cdc22-C11 ura4::adh-dmdNK-natMX-adh-hENT ura4-aim*
Eg3682	*h*$^-$ *leu1*$^+$*::nmt41-cdt2 Δcdt2::kanMX ura4::adh-dmdNK-natMX-adh-hENT cds1-2HA-his6::ura4*$^+$
Eg3683	*h*$^+$ *leu1*$^+$*::nmt41-cdt2 Δcdt2::kanMX ura4::adh-dmdNK-natMX-adh-hENT cds1-2HA-his6::ura4*$^+$ *Δspd1::hphMX*
Eg3706	*h*$^+$ *leu1*$^+$*::nmt41-cdt2 Δcdt2::kanMX Δrad3::ura4*$^+$ *ura4-D18::adh-suc22*$^+$
Eg3775	*h*$^+$ *leu1*$^+$*::nmt41-cdt2 Δcdt2::ura4+ ura4::adh-dmdNK-natMX-adh-hENT rad3-N2235K*
Eg3776	*h*$^+$ *leu1*$^+$*::nmt41-cdt2 Δcdt2::ura4+ ura4::adh-dmdNK-natMX-adh-hENT rad3-N2235K Δspd1::hphMX*
Eg3787	*h*$^+$ *rad3-N2235K leu1*$^+$*::nmt41-cdt2 Δcdt2::ura4*$^+$
Eg3788	*h*$^+$ *rad3-N2235K leu1*$^+$*::nmt41-cdt2 Δcdt2::ura4*$^+$ *Δspd1::hphMX*
Eg3837	*h*$^+$ *leu1*$^+$*::nmt41-cdt2 Δcdt2::kanMX ura4-D18 Δrad3::ura4*$^+$
Eg3838	*h*$^+$ *leu1*$^+$*::nmt41-cdt2 Δcdt2::kanMX ura4-D18 Δrad3::ura4*$^+$ *Δspd1::hphMX*
Eg3843	*h*$^+$ *leu1*$^+$*::nmt41-cdt2 Δcdt2::kanMX ura4-D18 Δrad26::ura4*$^+$
Eg3844	*h*$^+$ *leu1*$^+$*::nmt41-cdt2 Δcdt2::kanMX ura4-D18 Δrad26::ura4*$^+$ *Δspd1::hphMX*
Eg3845	*h*$^+$ *leu1*$^+$*::nmt41-cdt2 Δcdt2::kanMX ura4-D18 Δcds1::ura4*$^+$
Eg3846	*h*$^+$ *leu1*$^+$*::nmt41-cdt2 Δcdt2::kanMX ura4-D18 Δcds1::ura4*$^+$ *Δspd1::hphMX*
Eg3847	*h*$^+$ *leu1*$^+$*::nmt41-cdt2 Δcdt2::kanMX ura4-D18 Δmrc1::ura4*$^+$
Eg3848	*h*$^+$ *leu1*$^+$*::nmt41-cdt2 Δcdt2::kanMX ura4-D18 Δmrc1::ura4*$^+$ *Δspd1::hphMX*
Eg3858	*h*$^?$ *leu1*$^+$*::nmt41-cdt2 Δcdt2::kanMX Δchk1::ura4*$^+$
Eg3859	*h*$^?$ *leu1*$^+$*::nmt41-cdt2 Δcdt2::kanMX Δchk1::ura4*$^+$ *Δspd1::natMK*
Eg3851	*h*$^+$ *leu1*$^+$*::nmt41-cdt2 Δcdt2::kanMX ura4-D18 Δcrb2::ura4*$^+$
Eg3852	*h*$^+$ *leu1*$^+$*::nmt41-cdt2 Δcdt2::kanMX ura4-D18 Δcrb2::ura4*$^+$ *Δspd1::hphMX*
Eg3860	*h*$^?$ *leu1*$^+$*::nmt41-cdt2 Δcdt2::kanMX Δchk1::ura4*$^+$ *Δcds1::hphMX*
Eg3861	*h*$^?$ *leu1*$^+$*::nmt41-cdt2 Δcdt2::kanMX Δchk1::ura4*$^+$ *Δcds1::hphMX Δspd1::natMX*
Eg3835	*h*$^+$ *leu1*$^+$*::nmt41-cdt2 Δcdt2::kanMX ura4-D18 Δrad1::ura4*$^+$
Eg3836	*h*$^+$ *leu1*$^+$*::nmt41-cdt2 Δcdt2::kanMX ura4-D18 Δrad1::ura4*$^+$ *Δspd1::hphMX*
Eg3849	*h*$^+$ *leu1*$^+$*::nmt41-cdt2 Δcdt2::kanMX ura4-D18 Δrad9::ura4*$^+$
Eg3850	*h*$^+$ *leu1*$^+$*::nmt41-cdt2 Δcdt2::kanMX ura4-D18 Δrad9::ura4*$^+$ *Δspd1::hphMX*
Eg3839	*h*$^+$ *leu1*$^+$*::nmt41-cdt2 Δcdt2::kanMX ura4-D18 Δrad17::ura4*$^+$
Eg3840	*h*$^+$ *leu1*$^+$*::nmt41-cdt2 Δcdt2::kanMX ura4-D18 Δrad17::ura4*$^+$ *Δspd1::hphMX*
MG83	*h*$^+$ *ura4::adh::dmdNK-natMX-adh::hENT1 ura4-aim*
OL1144	*h*$^-$ *Δspd1::ura4 ura4-D18*
OL1368	*h*$^+$ *Δddb1::kanMX Δspd1::hphMX*
OL1687	*h*$^+$ *Δddb1::hphMX*
RO144	*h*$^-$ *smt-0*
